# Therapeutic combination of L-ascorbic acid, N-acetylcysteine, and dimethyl fumarate in Friedreich’s ataxia: insights from in vitro models

**DOI:** 10.1080/13510002.2025.2505303

**Published:** 2025-05-15

**Authors:** Fred Jonathan Edzeamey, Zenouska Ramchunder, Adamo Valle Gómez, Haobo Ge, Carlo Marya Thomas Marobbio, Charareh Pourzand, Sara Anjomani Virmouni

**Affiliations:** a Division of Biosciences, College of Health, Medicine, and Life Sciences (CHMLS), Institute of Environment, Health & Societies, Brunel University London, London, UK; b Energy Metabolism and Nutrition, Research Institute of Health Sciences (IUNICS), University of Balearic Islands, Palma, Spain; c Health Research Institute of Balearic Islands (IdISBa), Palma, Spain; d Biomedical Research Networking Center for Physiopathology of Obesity and Nutrition (CIBERobn CB06/03/0043), Instituto de Salud Carlos III, Madrid, Spain; e Department of Life Sciences, University of Bath, Bath, UK; f Centre for Therapeutic Innovation, University of Bath, Bath, UK; g Centre for Bioengineering and Biomedical Technologies, University of Bath, Bath, UK; h Department of Bioscience, Biotechnology and Environment, University of Bari, Bari, Italy

**Keywords:** Friedreich’s ataxia, FRDA, antioxidants, frataxin, oxidative stress, reactive oxygen species, neurodegeneration, mitochondrial dysfunction

## Abstract

Friedreich’s Ataxia (FRDA) is a rare neurological disorder caused by an abnormal expansion of Guanine-Adenine-Adenine (GAA) repeat in intron 1 of the *FXN* gene, which encodes frataxin, leading to reduced expression of frataxin, a mitochondrial protein essential for cellular homeostasis. Frataxin deficiency results in oxidative stress and mitochondrial dysfunction and impaired redox balance. Currently, there is no cure for FRDA. This study aimed to evaluate the therapeutic potential of antioxidants dimethyl fumarate (DMF), N-acetylcysteine (NAC), and L-ascorbic acid (LAA) in restoring mitochondrial redox homeostasis and frataxin levels in FRDA patient-derived fibroblasts and 2D sensory neurons. We assessed cell viability, mitochondrial and cellular reactive oxygen species (ROS) levels, mitochondrial DNA copy number, mitochondrial membrane potential, and frataxin and NRF2 expression at both mRNA and protein levels following antioxidant treatment, either individually or in combination. Treatment with LAA, NAC, and DMF resulted in significant reductions in mitochondrial and cellular ROS, along with increased FXN and NRF2 expression, and enhanced NRF2 nuclear translocation. Furthermore, these compounds improved aconitase/citrate synthase activity, GSH/GSSG ratios, and mitochondrial membrane potential. Notably, the combination of LAA and NAC consistently alleviated multiple disease-associated defects in FRDA cells, suggesting its potential as a promising therapeutic approach.

## 1. Introduction

Friedreich’s ataxia (FRDA) is a rare, lethal, autosomal recessive neurodegenerative disorder that affects approximately 1 in 50,000 Caucasians [1–4]. It is the most common hereditary ataxia, impacting the brain, spinal cord, heart, muscles, and pancreatic beta cells, leading to a variety of symptoms, including progressive neurodegeneration, musculoskeletal deformities, diabetes mellitus, and hypertrophic cardiomyopathy [2–4]. FRDA is caused by the abnormal expansion of the Guanine-Adenine-Adenine (GAA) repeat sequence in intron 1 of the *FXN* gene [3,4]. Approximately 96% of FRDA patients are homozygous for this GAA repeat expansion, while the remaining 4% have the expansion in one allele and a point mutation in the other [4]. This GAA repeat expansion leads to epigenetic changes, the formation of unusual DNA structures (such as sticky DNA and DNA/RNA hybrid structures), and increased heterochromatin formation. These alterations are hypothesised to cause reduced expression of the *FXN* gene and its protein product, frataxin [5,6]. Reduced frataxin levels are associated with mitochondrial iron overload, decreased ATP production, oxidative stress, downregulation of antioxidant response element (ARE)-regulated genes and subsequent cell death of sensory neurons in the dorsal root ganglia (DRG) and dentate nucleus [7–9].

To date, there is no cure for FRDA. Antioxidants have been investigated as potential therapeutic agents, with omaveloxolone (SKYCLARYS®) −a specific activator of the nuclear factor erythroid 2-related factor 2 (NRF2) − being the only approved drug, although its use has limitations [10]. NRF2 is a key regulator of the expression of antioxidant proteins, playing a crucial role in mitigating oxidative stress, a hallmark of FRDA [10]. Interestingly, oxidative stress itself can trigger NRF2 activation as an adaptive response to cellular damage, promoting the transcription of genes involved in antioxidant defence and mitochondrial function. Furthermore, NRF2 activation can be modulated by various natural compounds, including exogenous molecules such as polyphenols, flavonoids, chalcones and curcuminoids [11], as well as endogenous factors, with vitamin D emerging as a natural NRF2 inducer [12].

Beyond omaveloxolone, other antioxidants have also shown the ability to interact with the NRF2 pathway. N-acetylcysteine (NAC) exerts its antioxidant effects, at least in part, through NRF2 activation [13]. NAC hydrolysis leads to glutathione synthesis, thereby increasing glutathione levels, a key player of the antioxidant defence system [13]. In the context of FRDA, NAC has been studied in human fibroblasts derived from FRDA patients, where it significantly increases the expression of both *NRF2* and *FXN* genes [14]. However, its potential therapeutic effects in sensory neurons−one of the most affected cell types−remain unexplored.

Dimethyl fumarate (DMF) has been shown to activate the NRF2 pathway, leading to the induction of ARE-regulated genes and the mitigation of oxidative stress [15]. DMF (TECFIDERA®) has been approved by the European Medicines Agency (EMA) for the treatment of psoriasis and multiple sclerosis [10], and it has demonstrated a positive impact on frataxin expression in preclinical FRDA models [16,17]. However, its clinical application in FRDA has not been addressed.

L-ascorbic acid (LAA) exhibits its antioxidant activity by donating electrons to free radicals, thereby neutralising their reactivity. In addition to this, LAA is involved in the reduction of iron, which may have implications for iron homeostasis, and it activates hydroxylases [18]. Although LAA's roles in these processes are still not fully understood, its therapeutic potential in neurodegenerative diseases like FRDA has not been fully explored, either preclinically or clinically.

Given that FRDA is characterised by oxidative stress and LAA possesses antioxidant properties, we hypothesised that combining LAA with other antioxidants that have complementary mechanism of action could offer therapeutic benefits for FRDA. Additionally, combining commercially available drugs or antioxidants could provide cost-effective strategies with shorter clinical trial timelines. In this study, we explored the therapeutic effects of combining LAA with DMF and NAC in human FRDA fibroblasts and iPSC-derived 2D sensory neurons.

## 2. Materials and methods

### 2.1. Cell culture of human fibroblasts and viability studies

The healthy human fibroblast cell lines used in this study were GM23976 (Male, 22 years) and H-Normal (Female, 20 years). The FRDA human fibroblast cell lines included GM04078 (Male, 30 years, GAA 420/541), GM03816 (Female, 36 years, GAA 330/380) and FA-1 (GAA 416/590). GM23976, GM04078, and GM03816 were obtained from the Coriell Cell Repository (NJ, USA). H-Normal and FA-1 fibroblast cell line were generously provided by Dr Terry Roberts (Brunel University London, London, UK) and Dr Aurélien Bayot (Université Paris, France), respectively. FRDA and control fibroblast cell lines were maintained in DMEM (Invitrogen™) supplemented with 10% FBS (Invitrogen™) and 2% penicillin–streptomycin (Invitrogen™), at 37°C, 5% CO_2._ The compounds LAA (CAS: 50-81-7), NAC (CAS: 616-91-1) and DMF (CAS: 624-49-7) were all sourced from Sigma Aldrich. LAA and NAC were dissolved using deionised water, whilst DMF was dissolved in 0.1% DMSO.

For cell viability assays, 1 × 10^4^ cells were seeded into 96-well plates (CorningTM). After 24 hours, the medium was removed, and the cells were washed with PBS. They were then incubated for 72 hours with LAA at concentrations ranging from 10 µM to 440 µM, NAC from 0.5 mM to 27 mM, and DMF from 5 µM to 2000µM. Following treatment, the cells were washed with PBS, and pre-warmed medium containing 1X Presto-Blue® (cat#A13261, Invitrogen™) was added. The cells were incubated for an additional 3 hours at 37°C, 5% CO_2_, and 95% humidity. Absorbance was measured using a spectrophotometer (2000c, Invitrogen) at 570 nm with a 600 nm reference wavelength. PrestoBlue® reagent (Invitrogen) contains a non-fluorescent blue cell-permeant compound that is reduced by viable cells to produce a highly fluorescent red colour, which can be quantitively measured to determine cell viability.

### 2.2. Mitochondrial DNA (mtDNA) copy number

Mitochondrial DNA copy number was determined by extracting total DNA using phenol/chloroform extraction method, as previously described [19]. The mtDNA/nDNA ratio was then calculated using qPCR, following the protocol outlined by Jasoliya et al [16]. The forward and reverse primers used are listed in Table 1.

**Table 1.** Details of primers.

Species Gene	Sequence (5’→3’)
Human *FXN* Forward	TTGAAGACCTTGCAGACAAG
Human *FXN* Reverse	AGCCAGATTTGCTTGTTTGG
Human *NRF2* Forward	CACATCCAGTCAGAAACCAGTGG
Human *NRF2* Reverse	GGAATGTCTGCGCCAAAAGCTG
Human mt-TL1 (DNA) Forward	CACCCAAGAACAGGGTTTGT
Human mt-TL1 (DNA) Reverse	TGGCCATGGGTATGTTGTTA
Human B2M (DNA) Forward	TGCTGTCTCCATGTTTGATGTATCT
Human B2M (DNA) Reverse	TCTCTGCTCCCCACCTCTAAGT
Human BRN3A Forward	AGTACCCGTCGCTGCACTCCA
Human BRN3A Reverse	TTGCCCTGGGACACGGCGATG
Human HPRT Forward	GGTGAAAAGGACCCCACGA
Human HPRT Reverse	TCAAGGGCATATCCTACAACA

### 2.3. Quantitative real-time PCR (qRT-PCR)

RNA was extracted using the TRIzol/chloroform extraction method, followed by cDNA synthesis with QuantiTect Reverse Transcription Kit (cat#205311, QIAGEN). qRT-PCR was performed to assess *FXN* and *NRF2* gene expression, as previously described [19]. HPRT gene was used as the housekeeping gene. The forward and reverse primers used are listed in Table 1.

### 2.4. H_2_O_2_-induced toxicity

1 × 10^4^ cells were seeded into a 96 well plate (Corning^TM^) and incubated for 24 hours. The media was removed, and cells were washed with 1X PBS and this was followed by the incubation with the antioxidants for 72 hours. Cells were washed with 1X PBS and incubated with 1 mM H_2_O_2_ for 2 hours. The H_2_O_2_ was then removed, and the cells were incubated with medium for an additional 24 hours, followed by PrestoBlue™ Cell Viability assay, as previously described [20].

### 2.5. Measurement of mitochondrial reactive oxygen species (mROS), cellular ROS (cROS), ΔΨ_M,_ and mitochondrial mass by flow cytometry

#### 2.5.1. Measurement of mROS

Levels of mROS were determined using MitoSOX^TM^ Red (Cat#M36005) according to the manufacturer’s instructions. Briefly, 5.5 × 10^4^ cells were seeded into a 24-well plate (Corning^TM^) and incubated for 24 hours. The medium was then removed, and the cells were washed with 1X PBS before being treated with the respective drug concentrations: LAA (20, 220 and 440 µM), NAC (0.1, 1 and 2 mM), and DMF (30 and 50 µM) for 72 hours. As a positive control, cells were exposed to 5 µM Rotenone and Antimycin A (R/A) for 30 minutes. Following treatment, the medium was removed, and cells were incubated with 1 mM MitoSOX^TM^ Red (Thermo Fisher) for 30 mins. After incubation, cells were washed three times with Hank’s balanced salt solution (HBSS/Ca/Mg) and resuspended in fresh solution for analysis using a 488 laser flow cytometer (ACEA NovoCyte, Agilent Technologies, USA) with an excitation/emission of 510/580 nm. MitoSOX Red, a dihydroethidium derivative conjugated to a cationic triphenylphosphonium group, is a fluorogenic dye for the selective detection of mitochondrial superoxide in live cells. Within mitochondria, MitoSOX Red is selectively oxidised by superoxide, leading to the emission of red fluorescence. The intensity is proportional to mROS levels.

#### 2.5.2. Measurement of cROS

cROS levels were measured using H_2_DCFA (Cat#C400) following to the manufacturer’s instructions. Briefly, 5.5 × 10^4^ cells were seeded into a 24-well plate (Corning^TM^) and incubated for 24 hours. The medium was then removed, and cells were washed with 1X PBS before being treated with 20 µM LAA, 100 µM NAC and 30 µM DMF for 72 hours. As a positive control, cells were exposed to 5 µM R/A for 30 minutes. Following treatment, the medium was removed, and cells were incubated with 20 µM H_2_DCFA (Thermo Fisher) for 45 minutes. After incubation, cells were washed three times with HBSS and resuspended in fresh solution for analysis by flow cytometry with an excitation/emission of 485/535 nm. H_2_DCFA (2,7-dichlorodihydrofluorescein diacetate) is a non-polar, non-fluorescent compound that, upon oxidation by cROS, is converted into the highly fluorescent 2,7-dichlorofluorescein (DCF). The fluorescence intensity is directly proportional to cROS levels.

#### 2.5.3. Measurement of ΔΨ_M_

Mitochondrial membrane potential was assessed using TMRM (Cat#M20036) following the manufacturer’s instructions. Briefly, 5.5 × 10^4^ cells were seeded into a 24-well plate (Corning^TM^) and incubated for 24 hours. The medium was then removed, and the cells were washed with 1X PBS before being treated 20 µM LAA, 100 µM NAC and 30 µM DMF for 72 hours. As a negative control, cells were exposed to 20 µM of carbonyl cyanide-p-trifluromethoxyphenylhydrazone (FCCP) for 30 minutes. Following treatment, the medium was removed, and cells were incubated with 0.5nM TMRM reagent for 30 mins. Cells were then trypsinised, resuspended in DMEM, and analysed by flow cytometry (ACEA NovoCyte, Agilent Technologies, USA) with excitation/emission of 566/576 nm (phycoerythrin fluorescence channel).

#### 2.5.4. Measurement of Mitochondrial mass

Mitochondrial mass was measured using MitoTracker™ Green FM (Cat#M7514) according to the manufacturer’s instructions. Briefly, 5.5 × 10^4^ cells were seeded into a 24-well plate (Corning^TM^) and incubated for 24 hours. The media was then removed, and cells were washed with 1X prewarmed PBS. Following this, cells were treated 20 µM LAA, 100 µM NAC and 30 µM DMF for 72 hours. After treatment, cells were trypsinised and washed with 1X cold PBS. Cells were then resuspended in 50 µl of 100nM Mitotracker® Green and incubated at 37 ֯C for 15 mins. Finally, cells were resuspended in 300 µl PBS + 2%FBS for analysis using a 488-laser flow cytometer (ACEA NovoCyte, Agilent Technologies, USA) with an excitation/emission of 490/516 nm (FITC fluorescence channel).

### 2.6. Measurement of aconitase activity

Aconitase activity was measured using the Aconitase Assay Kit (cat#CAY705502, Cayman Chemical) according to the manufacturer’s instructions. The results were normalised to citrate synthase activity, determined using the Citrate Synthase Assay Kit (cat#CS0720, Sigma-Aldrich), according to the manufacturer’s instructions [19].

#### 2.7. Measurement of GSH/GSSG levels

Levels of GSH and GSSG in both FRDA and control fibroblasts following treatment were measured using the Glutathione Fluorescence Detection Kit (Cat#EIAGSHF, Invitrogen™) according to the manufacturer’s protocol. All experiments were performed in triplicate, and protein concentrations were determined using the Pierce™ BCA Protein Assay Kit (cat#23227, Thermo Scientific™) [19].

### 2.8. NRF2 immunofluorescence

Immunofluorescence was carried out as previously described [21]. Cells were incubated overnight at 4°C with anti-NRF2 antibody (1:100) (Invitrogen, Cat# PA5-27882), followed by incubation with Donkey anti-Rabbit IgG (H + L) highly cross-adsorbed secondary antibody, Alexa Fluor™ Plus 488 (1:2000) (Invitrogen Cat# A32790TR). Cells were then stained with DAPI and images were captured, and fluorescence intensity was determined using confocal microscopy. We analysed the original figures using Mender's coefficient with Image J software. An M1 value over 0.5 indicates strong colocalisation of Nrf2 with the DAPI signal, demonstrating strong nuclear colocalisation.

### 2.9. Culture, differentiation, and immunostaining of human FRDA and control iPSCs into 2D Sensory Neurons

Two FRDA, F4193 (clone 1; sex: female, age: 20 years, GAA repeat number:522/875) and F4041 (clone 5; sex: male, age: 19 years, GAA repeat number: 604/734), and two control, C6719 (sex: female, age: 22 years), and C3348 (sex: male, age:10 years), iPSC lines were used in this study. The iPSCs were maintained in culture in plates (Corning^TM^) coated with vitronectin (StemCell Technologies) in TeSR-E8 basal medium (StemCell Technologies). iPSCs were differentiated into 2D sensory neurons and immunoassayed with anti-BRN3A and anti-Peripherin, following the protocol described by Viventi and colleagues [22]. After differentiation, the sensory neurons were treated with LAA and NAC for subsequent assays.

### 2.10. Frataxin and NRF2 protein quantification using ELISA

Frataxin and NRF2 protein levels in both FRDA and control human fibroblast cells as well as in sensory neurons were assessed using human frataxin ELISA kit (cat#ab176112, Abcam) and Human NRF2 ELISA kit (cat#ab277397, Abcam), according to the manufacturer’s instructions. All experiments were performed in triplicate, and protein concentrations were determined as previously described [19].

### 2.11. Analysis of Flow cytometry data

Gating was done using an Untreated, unstained control cell line. Cells were stained with the appropriate probe and the respective channel was used for the excitation and emission of the probe. The light scatter: Forward scatter (FS) vs Side scatter (SS) was used to select the desired population of cells whose fluorescence intensity were quantified. Dead cells were excluded from the analysis.

### 2.12. Statistical analysis

Statistical tests were performed using GraphPad Prism (v9.0). An unpaired, two-tailed Student’s *t*-test was used to assess significant differences between group data, with significance set at *P* < 0.05. For comparisons involving two or more treatment groups, one-way ANOVA followed by Tukey’s multiple comparisons test was utilised, with significance set at *P* < 0.05.

## 3. Results

### 3.1. Cell viability assays and mROS reducing capacity of antioxidants

**Figure 1.**
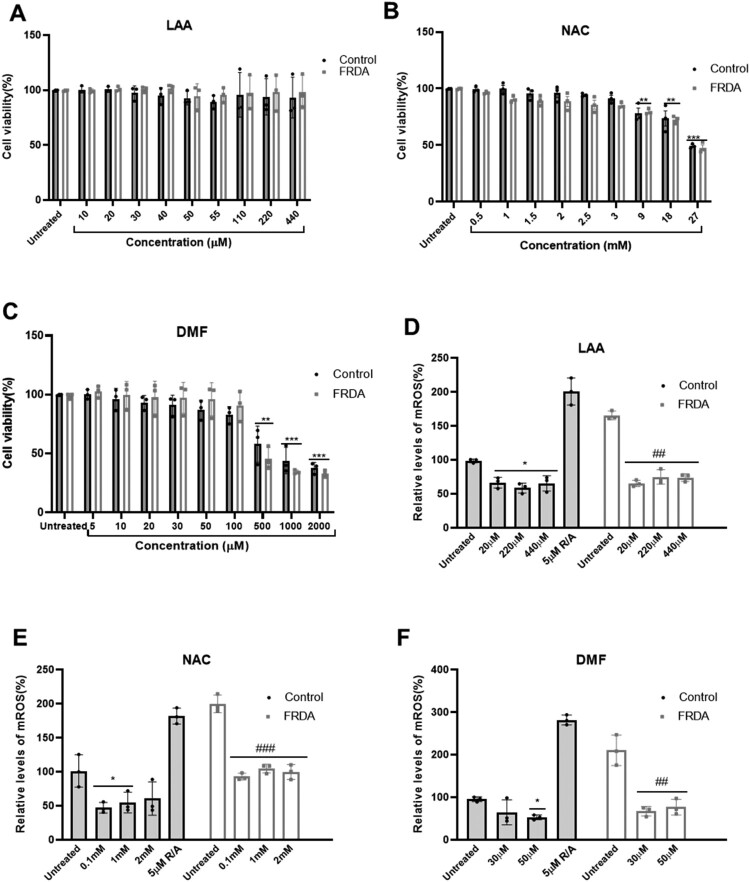
Cell viability studies and mROS-reducing capacity of compounds using MitoSOX Red dye in human FRDA and control fibroblast cell lines. Cell viability was assessed following treatment with (A) L-Ascorbic acid (LAA), (B) N-acetyl cysteine (NAC), and (C) Dimethyl fumarate (DMF) for 72 hours. The mean value of all data set was normalised to the PrestoBlue® reduction of the vehicle (pegged at 100%). Five human fibroblasts cell lines were used, comprising two controls and three FRDA lines. All data are presented as mean ± SEM from three independent experiments. Significant differences between the treated and untreated groups were analysed using two-way ANOVA with Bonferroni’s post hoc test and indicated by * where (**P < 0.01, ***P < 0.001, comparison between vehicle and treated of each group). mROS reducing capacity of (D) LAA, (E) NAC, and (F) DMF treatments for 72 hours. The mean value of all data set was normalised to the untreated group of control (pegged at 100%). All data are presented as mean ± SEM from three independent experiments. Significant differences between the treated and untreated groups were analysed using two-way ANOVA with Bonferroni’s post hoc test and indicated by * and # where (*P < 0.05, **P < 0.01, ***P < 0.001, comparisons against untreated control vs treated control), (##*P* < 0.01, ### *P* < 0.001, untreated FRDA vs treated FRDA).

To evaluate the potential effects of antioxidants on mROS, we first determined the optimal concentrations of LAA, NAC, and DMF though cell viability studies, evaluating the safety and cellular tolerability of different concentrations of these compounds. For LAA, concentrations ranging from 10 µM to 440 µM were tested, with no detectable cellular toxicity. For NAC, doses between 0.5 mM to 27 mM were assessed, and cells exhibited tolerance at concentrations below 1 mM. In the case of DMF, concentrations from 0.5 µM to 2000µM were tested, and cells tolerated doses below 500 µM (Figure 1A-C). These concentrations were subsequently used to assess mROS levels in FRDA fibroblasts, where all three compounds were found to significantly reduce mROS levels (Figure 1D-F). The cellular toxicity and mROS reducing capacity of the compounds, as recorded in individual cell line, are presented in supplementary Figure 1A-F.

### 3.2. Antioxidants increase FXN and NRF2 gene expression in human FRDA fibroblasts

**Figure 2.**
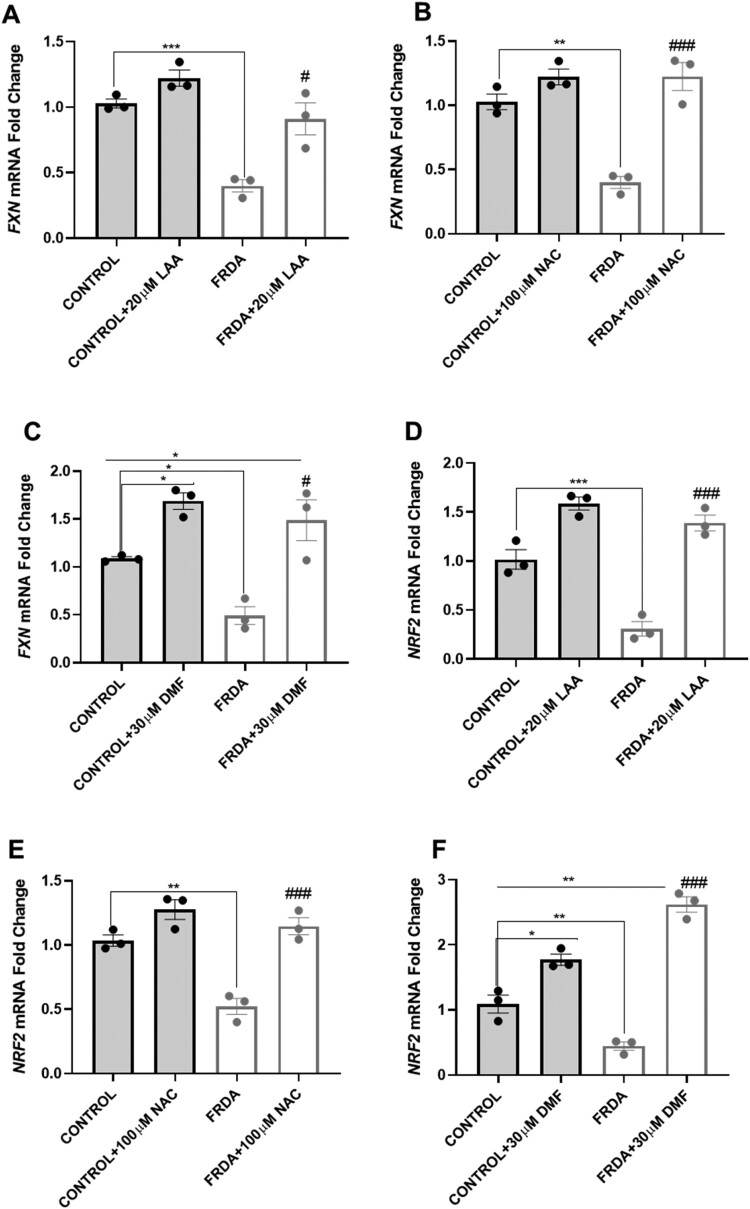
Treatment with LAA, NAC, and DMF led to significant increases in *FXN* and *NRF2* gene expression in human FRDA fibroblast cell lines. Effect of (A) LAA, (B) NAC, (C) DMF treatments for 72 hours on *FXN* gene expression levels. Effect of (D) LAA, (E) NAC and (F) DMF treatments for 72 hours on *NRF2* gene expression levels. Five human fibroblasts cell lines were used, comprising two controls and three FRDA lines. The mean value of all data set, from at least three independent experiments, normalised to the untreated groups of control (pegged at 1). All data are presented as mean ± SEM. Significant differences between the treated and untreated group were analysed using Student’s *t*-test and indicated by * and # where (***P < 0.001, Comparisons against untreated control), (# *P* < 0.05, ## *P* < 0.01, ### *P* < 0.001, untreated FRDA vs treated FRDA).

The low levels of *FXN* gene expression have been identified as a result of the abnormal GAA repeat sequence in intron 1 of the *FXN* gene, a genetic aberration that drives disease progression. Additionally, *NRF2*, a transcription factor involved in the expression of antioxidant enzymes, is observed to be downregulated in FRDA patients. To address this, we investigated the effect of antioxidants on the mRNA expression levels of *FXN* and *NRF2* in fibroblast cells using qPCR. For each antioxidant, we selected the lowest concentration that significantly reduced mROS (Figure 1D-F) to optimise the efficacy/safety balance, minimising the risk of non-specific effects or excessive alterations of redox-sensitive pathways. Treatment with 20 µM LAA, 100 µM NAC and 30 µM DMF resulted in a significant increase in mRNA expression levels of both *FXN* (Figure 2A-2C) and *NRF2* (Figure 2D-2F). Interestingly, these compounds were able to increase *FXN* and *NRF2* gene expression to levels comparable to those observed in control fibroblasts. The differential expression of *FXN* and *NRF2* genes in individual fibroblast cell lines is shown in supplementary Figure 2A-F.

### 3.3. Therapeutic combination of LAA, NAC and DMF improves FRDA phenotypes in human FRDA fibroblast cell lines

**Figure 3.**
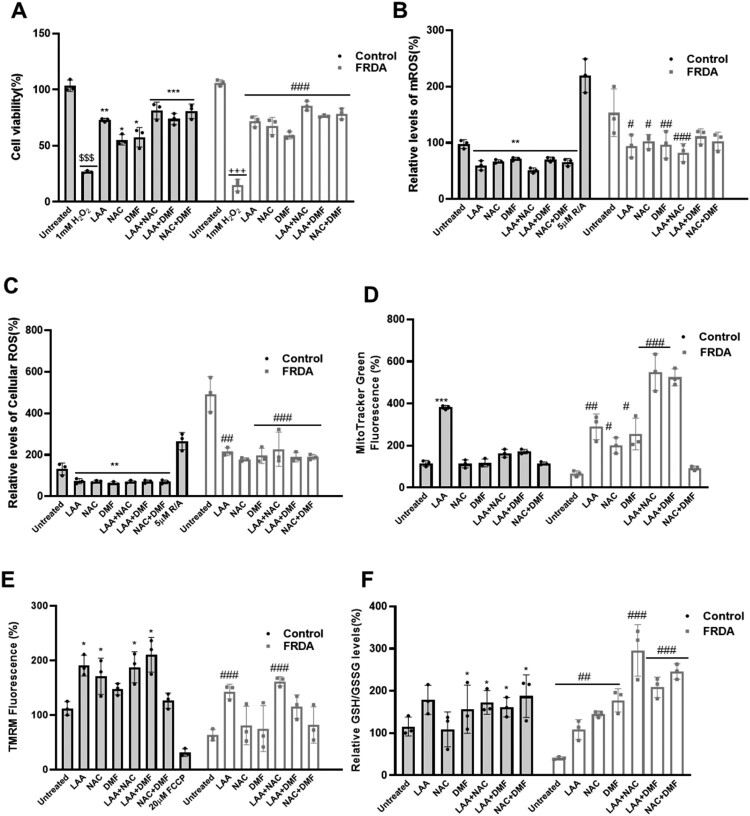
Effect of antioxidant combination on oxidative stress and antioxidant markers in human FRDA fibroblast cell lines. (A) Protection against H_2_O_2_-induced oxidative toxicity by antioxidant combinations in human FRDA and control fibroblasts. Significant differences between the treated and untreated groups were analysed using one-way ANOVA with Tukey’s multiple comparisons test, indicated by $, +, * and # where ($$$ *P* < 0.001, untreated Control vs Control + H_2_O_2_), (+++ *P* < 0.001, untreated FRDA vs FRDA + H_2_O_2_), (*P < 0.05, **P < 0.01, ***P < 0.001, control + H_2_O_2_ vs control pre-treated with antioxidants + H_2_O_2_), (### *P* < 0.001, FRDA + H_2_O_2_ vs FRDA pretreated with antioxidants + H_2_O_2_). Concentrations of antioxidants used were 20 µM for LAA, 100 µM for NAC and 30 µM DMF. Antioxidant combinations’ impact on (B) mROS, (C) cROS, (D) mitochondrial mass, (E) ΔΨ_M_, and (F) GSH/GSSG ratio. Significant differences between the treated and untreated groups were analysed using one-way ANOVA with Tukey’s multiple comparisons test, indicated by $, * and # where (*P < 0.05, **P < 0.01, ***P < 0.001 untreated control vs control treated with antioxidants), (# *P* < 0.05, ## *P* < 0.01, ### *P* < 0.001, FRDA untreated vs FRDA treated with antioxidants). Five human fibroblasts cell lines were used, comprising two controls and three FRDA lines The mean value of all data set was normalised to the untreated group (set at 100%). All data are presented as mean ± SEM from three independent experiments.

Following the positive outcomes with LAA, NAC, and DMF individually, we explored the effects of the therapeutic combination of these compounds; LAA + NAC, LAA + DMF, and NAC + DMF. FRDA and control cells were pre-treated with these combinations for 72 hours, followed by exposure to 1 mM H_2_O_2_ for 2 hours. After H_2_O_2_ treatment, the medium was replaced, and the cells were incubated for an additional 24 hours before assessing cell viability using PrestoBlue™ Cell Viability Assay. As expected, H_2_O_2_-induced a significant reduction in cell viability (*P* < 0.001) in the untreated cell lines (Figure 3). In the pre-treated cells, all antioxidant treatments provided significant protection against the oxidative toxicity induced by H_2_O_2_ (Figure 3A). Amongst the combinations, LAA + NAC exhibited the highest protective effect in nominal terms although there was no statistical difference between the three combinations (Figure 3A). Following experiments to identify protective effects of the compounds against H_2_O_2_-induced oxidative toxicity, the effect of the compounds on mitochondrial function was investigated. For ROS experiments, 5 µM R/A was used as a positive control to induce ROS via electron transport chain and oxidative phosphorylation disruption. As anticipated, treatment with R/A in healthy fibroblast cell lines resulted in increased mROS and cROS levels, thus validating the results identified with the compounds. LAA + NAC resulted in a significant reduction in mROS levels (*P* < 0.001) (Figure 3B). All treatments significantly reduced the levels of cROS (Figure 3C). We have also observed an increase in the level of mitochondrial mass using MitoTracker Green in the FRDA cells treated with LAA + NAC and LAA + DMF (*P* < 0.001) (Figure 3D). Moreover, LAA + NAC was shown to be the most effective combination for increasing ΔΨ_M_ (*P* < 0.001) (Figure 3E) and GSH/GSSG ratio in FRDA fibroblasts (*P* < 0.001) (Figure 3F). 20 µM FCCP was used as a negative control for ΔΨ_M_ experiments. As expected, treatment with this oxidative phosphorylation uncoupler led to reduced ΔΨ_M_ in healthy fibroblast cell lines, further validating the results obtained with regards to mitochondrial function. We also observed a significant increase in total glutathione following the treatment with the compounds (Supp Fig 3F). The effects of the therapeutic combination, as recorded in individual human fibroblast cell lines, are shown in supplementary Fig 3A-E.

### 3.4. Antioxidant treatments increased mitochondrial DNA copy number in human FRDA fibroblasts.

**Figure 4.**
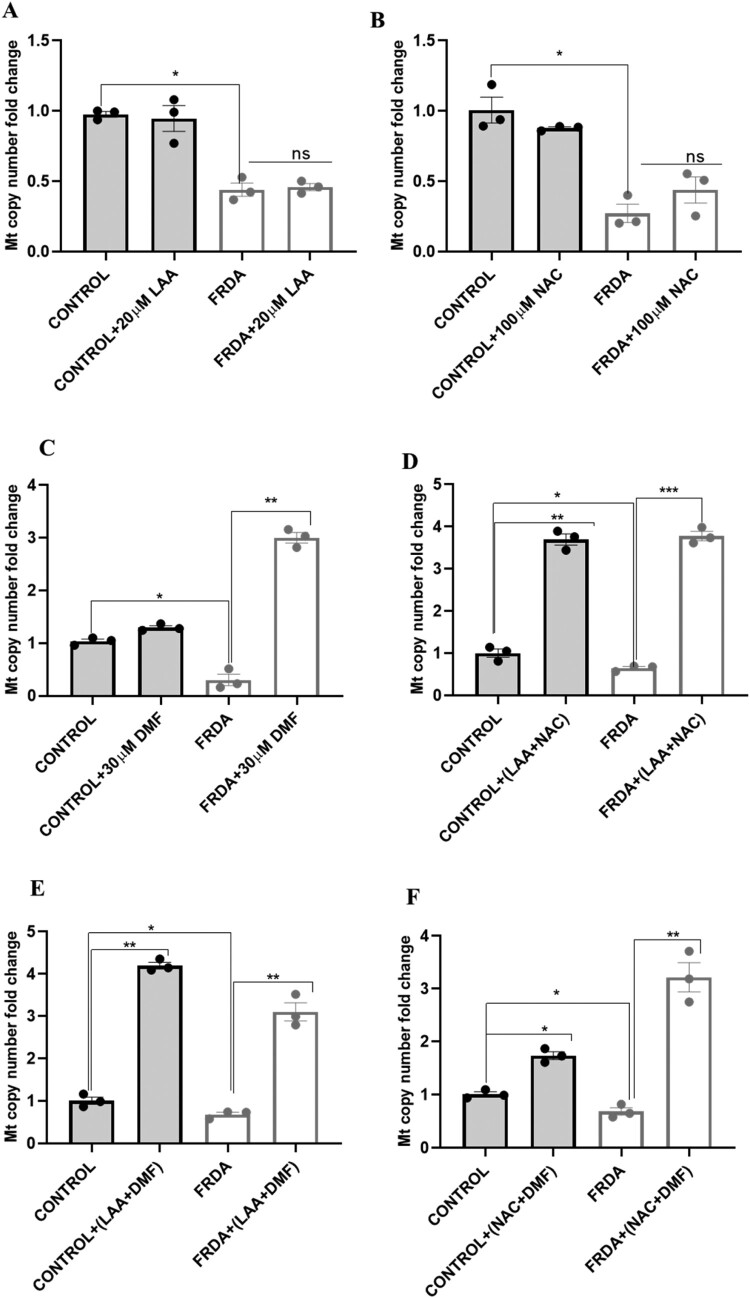
Mitochondrial DNA copy number analysis in FRDA fibroblasts following treatment with antioxidants. qPCR analysis of mtDNA/nDNA ratio after 72 hours treatment with (A) LAA, (B) NAC, (C) DMF, (D) LAA + NAC, (E) LAA + DMF, and (F) NAC + DMF. Concentrations of antioxidants used were 20 µM for LAA, 100 µM for NAC and 30 µM DMF. Five human fibroblasts cell lines were used, comprising two controls and three FRDA lines. The mean value of all data set, from three independent experiments, was normalised to the untreated groups of control (pegged at 1). All data are presented as mean ± SEM. Significant differences between the treated and untreated groups were analysed using Student’s *t*-test (ns = not significant, *P < 0.05, **P < 0.01, ***P < 0.001).

LAA and NAC, when used as monotherapies, did not significantly alter mitochondrial copy number (Figure 4A-B). In contrast, DMF treatment resulted in a significant increase in mitochondrial copy number (Figure 4C). Notably, the combinations of LAA + NAC (Figure 4D), LAA + DMF (Figure 4E), and NAC + DMF (Figure 4F) all significantly increased mitochondrial DNA copy number in both FRDA and control fibroblasts. In supplementary Figure 4A-F, we report the changes in the mitochondrial copy number in individual human fibroblast cell line.

### 3.5. Antioxidant compounds increased frataxin and NRF2 protein levels, aconitase activity, and NRF2 nuclear translocation

**Figure 5.**
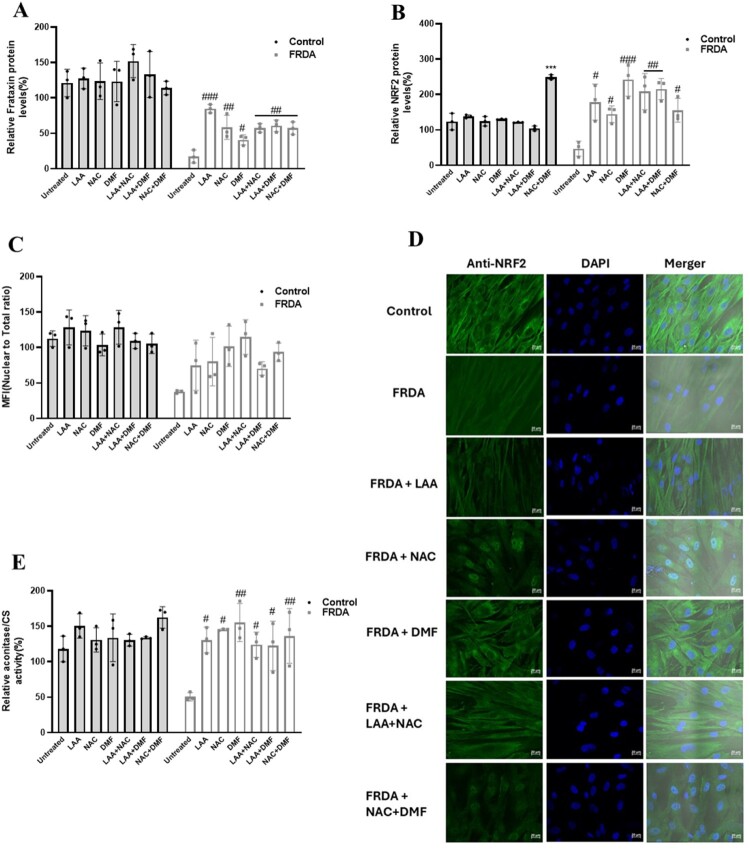
Effect of antioxidants on frataxin and NRF2 protein expression levels, aconitase activity and NRF2 nuclear translocation. Effect of antioxidants on (A) frataxin protein expression levels, (B) NRF2 protein levels, (C) aconitase activity, (D) NRF2 nuclear translocation visualised by confocal microscopy using anti-NRF2 antibody (green) and DAPI (blue), and (E) quantification of NRF2 fluorescence. Five human fibroblasts cell lines were used, comprising two controls and three FRDA lines. Data represent the mean of three replicates, normalised to the untreated control group (set at 100%). All data are presented as mean ± SEM. Significant differences between treated and untreated groups were analysed using one-way ANOVA with Tukey’s multiple comparisons test, indicated by * and # where (** *P* < 0.01, *** *P* < 0.001, untreated FRDA vs untreated control), (# *P* < 0.05, ## *P* < 0.01, ### *P* < 0.001, untreated FRDA vs FRDA treated with antioxidants).

From the gene expression data, we observed a downregulation *FXN* and *NRF2* genes in FRDA patients. Also, low levels of the important mitochondrial protein frataxin have been reported to be the main driver of disease pathogenesis in FRDA. Furthermore, reduced levels of NRF2 protein have been reported in FRDA. We therefore assessed the changes in frataxin and NRF2 protein levels using ELISA and/or Western blot analysis following the treatment with the antioxidants. Aconitase and citrate synthase are important enzymes of the citric acid cycle required for mitochondrial bioenergetics. Aconitase activity has been reported to be impaired by oxidative stress inducers such as hydrogen peroxide, superoxide ions and other peroxyl radicals. The accumulation of these radicals has been well documented in FRDA. We therefore assessed the levels of aconitase activity by normalising to citrate synthase activity to determine the effect of the antioxidants in human FRDA fibroblasts. Treatment of the FRDA fibroblasts with the individual compounds and their combinations resulted in significant increases in frataxin and NRF2 expression levels (Figure 5A-B). We also observed an increased in frataxin protein levels using western blot analysis (Supp Fig 6A-B). Additionally, we observed increased aconitase activity (Figure 5E), citrate synthase activity (Supp Fig 5D). and enhanced NRF2 nuclear translocation in the FRDA cells (Figure 5C-D) (Supp Fig 6C-E). Supplementary Fig 5A-D depict the result in individual human fibroblast cell line.

### 3.6. Characterisation of 2D sensory neurons and therapeutic effect of LAA + NAC treatment on FXN and NRF2 mRNA levels, and Frataxin and NRF2 protein levels in FRDA-derived 2D Sensory neurons

**Figure 6.**
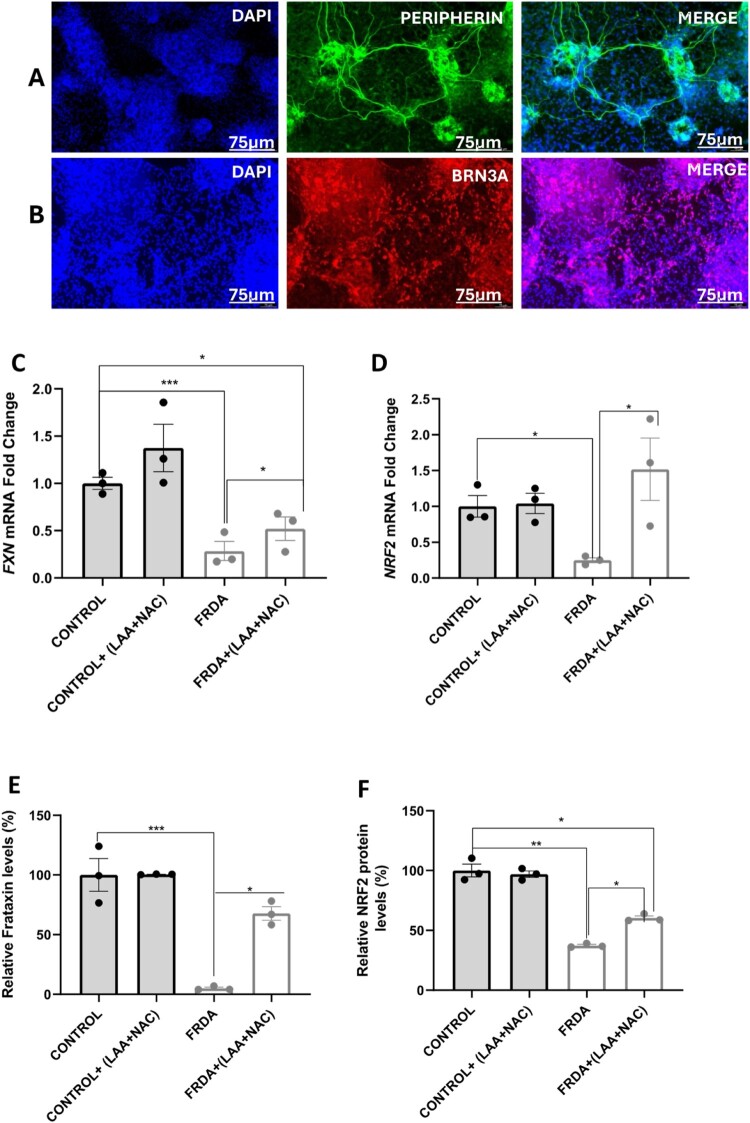
Therapeutic effects of antioxidants in 2D sensory neurons. Characterisation of sensory neurons and Effect of LAA + NAC on frataxin and NRF2 gene and protein expression levels in 2D sensory neurons. Micrographs of immunofluorescence staining for sensory neuron markers (A) Peripherin and (B) BRN3A. Images were captured using a Leica MD4000 Microscope. Blue indicates DAPI, green marks Peripherin, and red marks BRN3A. Effect of LAA + NAC on (C) *FXN* and (D) *NRF2* gene expression levels. Data from three independent experiments were normalised to the untreated control group (set at 1). Effect of LAA + NAC on (E) frataxin and (F) NRF2 protein expression levels. Data from three independent experiments were normalised to the untreated control group (pegged at 100%). Four human iPSC-derived 2D sensory neurons lines were used, comprising two control and two FRDA lines. All data are presented as mean ± SEM. Significant differences between the treated and untreated groups were analysed using Student’s t-test (*P < 0.05, **P < 0.01, ***P < 0.001).

Given the effectiveness of the 20 µM LAA + 100 µM NAC combination in targeting mitochondrial dysregulation in FRDA fibroblasts, we extended our assessment to its therapeutic effect in control and FRDA iPSC-derived 2D sensory neurons. The 2D sensory neurons were characterised using antibodies against sensory neuron markers (Anti-BRN3A and Anti-Peripherin) (Figure 6A-B). As shown in Figure 6C and D, treatment with LAA + NAC significantly increased *FXN* and *NRF2* mRNA expression levels in FRDA 2D sensory neurons (*P* < 0.05). A similar trend was also observed at the protein level (Figure 6E-F). The effects of LAA + NAC on individual iPSC-derived 2D sensory neuron are depicted in supplementary Fig 7A-D.

### 3.7. LAA + NAC increased mitochondrial copy number and reduced mROS levels in FRDA-derived 2D sensory neurons

**Figure 7.**
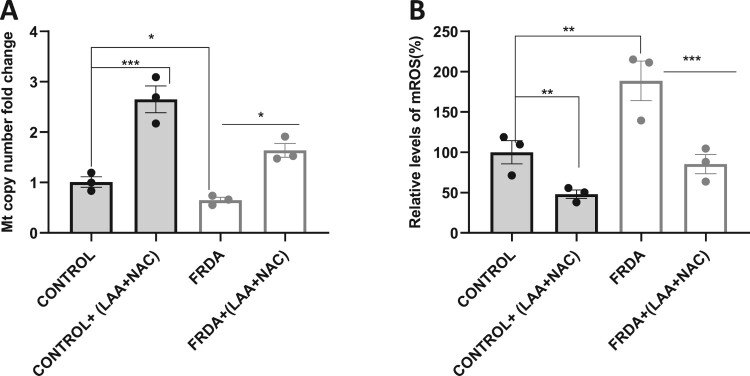
LAA + NAC increased mitochondrial copy number and reduced mROS 2D sensory neurons. (A) Mitochondrial DNA copy number following qPCR analysis of mtDNA/nDNa ratio after 72 hours treatment with LAA + NAC. Data from three independent experiments were normalized to the untreated control group (set at 1). (B) Assessment of mROS using MitoSOX Red dye in 2D sensory neurons derived from human FRDA iPSCs after treatment with LAA + NAC for 72 hours. The mean value of all data set, from three independent experiments, was normalised to the untreated groups of control (pegged at 100%). Four human iPSC-derived 2D sensory neurons lines were used, comprising two control and two FRDA lines. All data are presented as mean ± SEM. Significant differences between the treated and untreated group were analysed using Student’s *t*-test (*P < 0.05, **P < 0.01, ***P < 0.001).

A significant increase in mitochondrial DNA copy number (*P* < 0.05) was observed in 2D sensory neurons following the treatment with 20 µM LAA + 100 µM NAC (Figure 7A). With the same treatment, we observed a significant reduction in mROS levels (*P* < 0.001) in both in FRDA and control 2D sensory neurons (Figure 7B). The effects of LAA + NAC on individual iPSC-derived 2D sensory neuron are depicted in supplementary Fig 7E-F.

## 4. Discussion

FRDA is a lethal autosomal neurodegenerative disorder caused by the abnormal expansion of the GAA repeat sequence in intron 1 of the *FXN* gene [4,19]. This expansion leads to the downregulation of the *FXN* gene and a consequent reduction in the production of frataxin, a critical mitochondrial protein involved in iron homeostasis [4,19]. Low frataxin levels have been reported to be the primary driver of disease progression, making frataxin restoration a key therapeutic target. The downstream consequences of frataxin deficiency include mitochondrial iron accumulation, impaired mitochondrial bioenergetics and ATP synthesis, and increased oxidative stress leading to apoptosis [23,24]. Also, iron-induced cell death (ferroptosis) has been reported in preclinical FRDA models where increased sensitivity to ferroptosis inducer was recorded in FRDA models when compared to normal control cells [25,26]. The FRDA cells were observed to be rescued from ferroptosis following the treatment with ferroptosis inhibitor SRS11-92. This further indicates the consequences of iron accumulation in the mitochondria of FRDA patients [26]. In addition, FRDA patients exhibit significant downregulation of endogenous antioxidant defence systems, including NRF2, a multifunctional transcription factor [14]. The identification of oxidative stress coupled with the downregulation of antioxidant defences in FRDA has driven significant interest in exploring as potential therapeutic interventions in the disease. Motivated by the well-established role of oxidative stress in FRDA pathology, we conducted this study to evaluate the therapeutic effects of antioxidants (NAC, DMF and LAA) in FRDA using human fibroblast cell lines and iPSC-derived 2D sensory neurons obtained from FRDA and healthy patients. Human fibroblast cell lines obtained from FRDA patients have been extensively studied and are considered a reliable *in vitro* model for testing potential FRDA therapies. These cell lines exhibit characteristic features of the disease, including GAA expansion, reduced *FXN* gene expression, frataxin deficiency, increased oxidative stress, and downregulation of antioxidant defences, including reduced NRF2 expression at both gene and protein levels [14,27].

Given this, we first determined the non-toxic concentrations of each compound that effectively reduced mROS levels, as measured by the MitoSOX Red probe, in the fibroblast cell lines. All three compounds -LAA, NAC and DMF- significantly reduced mROS levels, a key pathological feature of the disease [23], in FRDA cells. This reduction in mROS indicated the potential therapeutic efficacy of the compounds. We further assessed their effect on *FXN* and *NRF2* gene expression in both human FRDA and control fibroblasts, finding a significant upregulation of both genes following treatment with LAA, NAC, and DMF. While the effects of LAA on FXN and NRF2 gene expression in FRDA cells had not been previously reported, our results for NAC and DMF align with earlier studies that demonstrated increased *NRF2* and *FXN* mRNA expression in FRDA fibroblasts treated with these antioxidants [16,17,28]. The increase in *FXN* gene expression following antioxidant treatment has been attributed to the activation of AREs, which have been reported to be present at the transcription start site of the *FXN* locus [14,29]. This suggests that the compounds activate *NRF2*, which in turn stimulates the AREs, leading to increased *FXN* expression [30]. Therefore, we propose that the observed upregulation of *FXN* gene expression is likely due to the activation of the *NRF2* pathway, which induces the activation of AREs at the *FXN* transcription start site.

Combination therapies are often used to enhance treatment outcomes. With this in mind, we investigated the effects of LAA+NAC, LAA+DMF, and NAC+DMF on various pathological features of FRDA. Among these, the LAA+NAC combination demonstrated the most effective protection against H_2_O_2_-induced oxidative stress. This combination consistently yielded significant benefits, including restoring frataxin and NRF2 protein levels, increasing mitochondrial mass, reducing both mROS and cROS, preventing mitochondrial membrane potential collapse, enhancing NRF2 nuclear translocation, and increasing mitochondrial DNA copy number, as well as aconitase/citrate synthase activity, and GSH/GSSG ratios in FRDA fibroblast cells. NAC is a known activator of NRF2, leading to the upregulation of genes such as HO-1 [14,31], while LAA acts as a potent ROS scavenger [32]. The complementary nature of their antioxidant effects suggests that the combined impact of LAA and NAC in FRDA fibroblasts holds substantial therapeutic potential.

Although patient-derived skin fibroblasts are widely recognised as a suitable *in vitro* model for studying FRDA and effectively replicate many of characteristic features of the disease, it is important to note that they are not the most severely affected cell type in these patients. Consequently, utilising other cell types may offer more precise insights into the efficacy of treatments. Notably, DRG neurons are among the most significantly impacted cell types in FRDA. These sensory neurons, located in the DRG, play a vital role in transmitting sensory information essential for proprioception and coordination. Their degeneration markedly contributes to the hallmark ataxia and sensory deficits observed in the disease. To investigate whether the combination of LAA and NAC can ameliorate the characteristic alterations associated with FRDA in this cell type, we differentiated 2D sensory neurons from iPSCs derived from both FRDA patients and healthy controls.

In agreement with previous studies [33,34], our iPSC-derived sensory neurons from FRDA patients accurately reflected the disease phenotype, exhibiting increased levels of mROS, reduced mitochondrial copy number, and decreased mRNA and protein levels of frataxin and NRF2 compared to sensory neurons derived from healthy individuals. Notably, treatment of the FRDA sensory neurons with LAA and NAC resulted in a significant reduction in mROS levels, along with increase in both FXN gene and protein levels, as well as enhanced NRF2 gene and protein expression.

Our findings indicate that antioxidant treatment induces frataxin expression, prompting the question of how antioxidants enhance the expression of this gene. This effect has been previously attributed to the presence of AREs at the transcription start site of the *FXN* locus [14]. This suggest that the antioxidant compounds activate *NRF2,* which subsequently activates AREs, leading to increased *FXN* expression [30]. While our data show the upregulation of FXN expression in response to antioxidant treatment, we did not analyse the nuclear translocation of NRF2 in sensory neurons in this study. However, we did observe an increase in NRF2 levels, both at the gene and protein levels, in response to the treatment. The nuclear localisation of NRF2 is indeed an important aspect to fully understand its role in the activation of AREs and the regulation of genes like *FXN*. Therefore, we propose that the observed upregulation of *FXN* gene expression could result from the activation of the *NRF2* pathway, which induces the activation of AREs at the *FXN* transcription start site. Further experiments, including the analysis of NRF2 nuclear translocation in sensory neurons, will be necessary to confirm that antioxidants can exert a dual effect, acting both as scavengers and by inducing antioxidant proteins under the regulation of NRF2, including frataxin itself.

In conclusion, our study demonstrates the therapeutic potential of antioxidants in FRDA, using two different *in vitro* models: human FRDA fibroblasts and 2D sensory neurons derived from FRDA-derived iPSCs. The combination of LAA and NAC emerged as the most promising treatment, demonstrating a robust therapeutic profile in both cell types. These findings suggest that LAA+NAC is a strong candidate for further investigation and potential clinical translation in FRDA therapy. To strengthen the translational relevance of these results, the efficacy and toxicity of these antioxidants should be further evaluated in relevant *in vivo* models used to study oxidative stress and drug screening [3,19,35,36]. These next steps will be essential for validating both the safety and therapeutic potential of the compounds before progressing to clinical trials.

## Supplementary Material

Supplemental Material

## Data Availability

The data used to support the findings of this study are available from the corresponding author upon request.
