# Supplementary material for: Therapeutic combination of L-ascorbic acid, N-acetylcysteine, and dimethyl fumarate in Friedreich’s ataxia: insights from in vitro models
Source: Redox Rep. 2025 May 15;30(1):2505303. doi: 10.1080/13510002.2025.2505303 (PMC12082744; doi:10.1080/13510002.2025.2505303)
Supplement: Supplemental Material [file YRER_A_2505303_SM5461.docx]

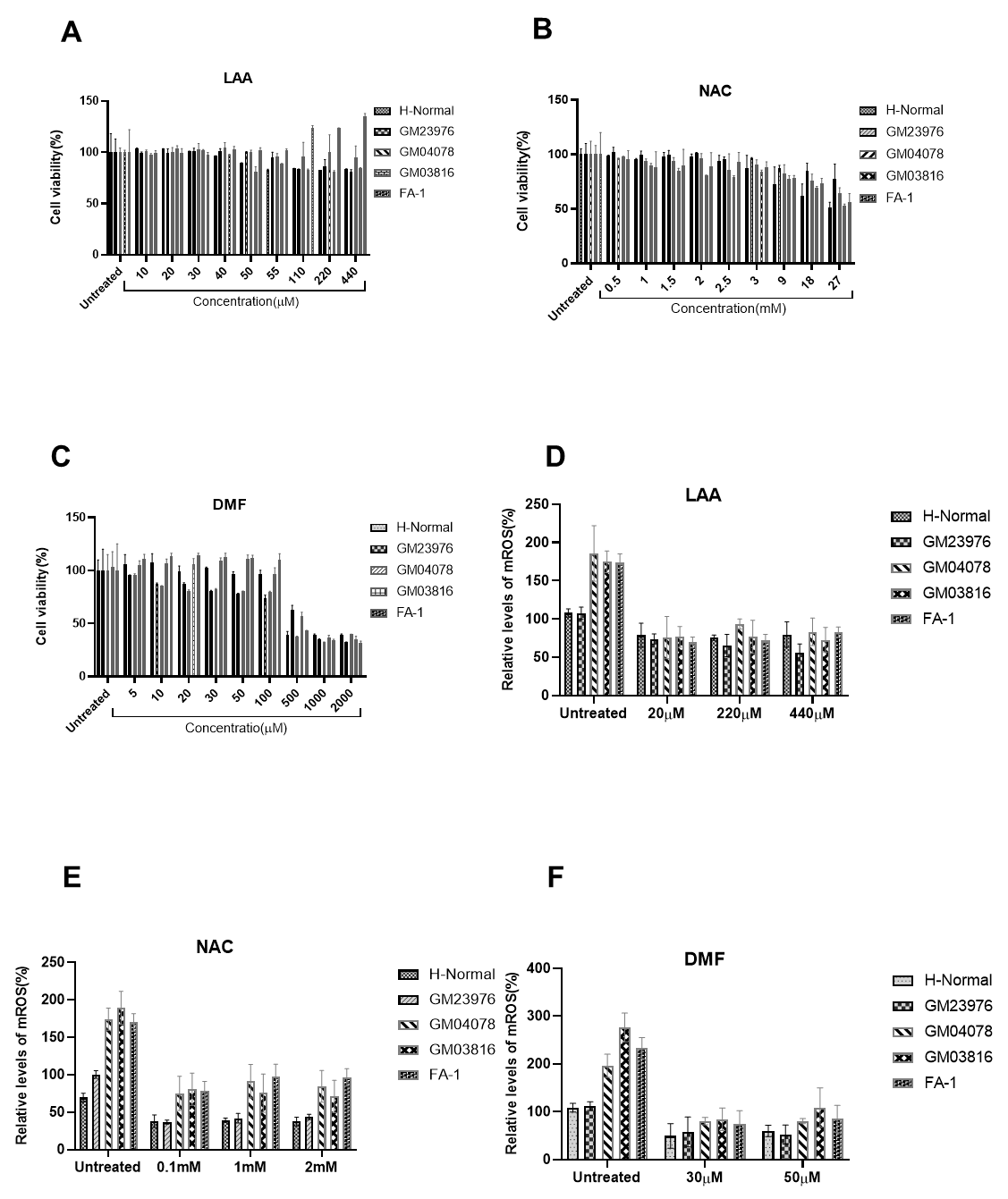
**Supplemental material**

**Supplementary Figure 1. Cell viability studies and mROS-reducing capacity of compounds using MitoSOX Red dye in human control and FRDA fibroblast cell lines.** Cell viability was assessed following treatment with (A) L-Ascorbic acid (LAA), (B) N-acetyl cysteine (NAC), and (C) Dimethyl fumarate (DMF) for 72 hours. The mean value of all data set was normalised to the PrestoBlue® reduction of the Untreated (pegged at 100%). Five human fibroblasts cell lines were used, comprising two controls (H-Normal, GM23976) and three FRDA lines (GM04078, GM03816, FA-1). All data are presented as mean±SEM from three independent experiments. mROS reducing capacity of (D) LAA, (E) NAC, and (F) DMF treatments for 72 hours. The mean value of all data set was normalised to the untreated group of control (pegged at 100%). All data are presented as mean±SEM from three independent experiments.

**
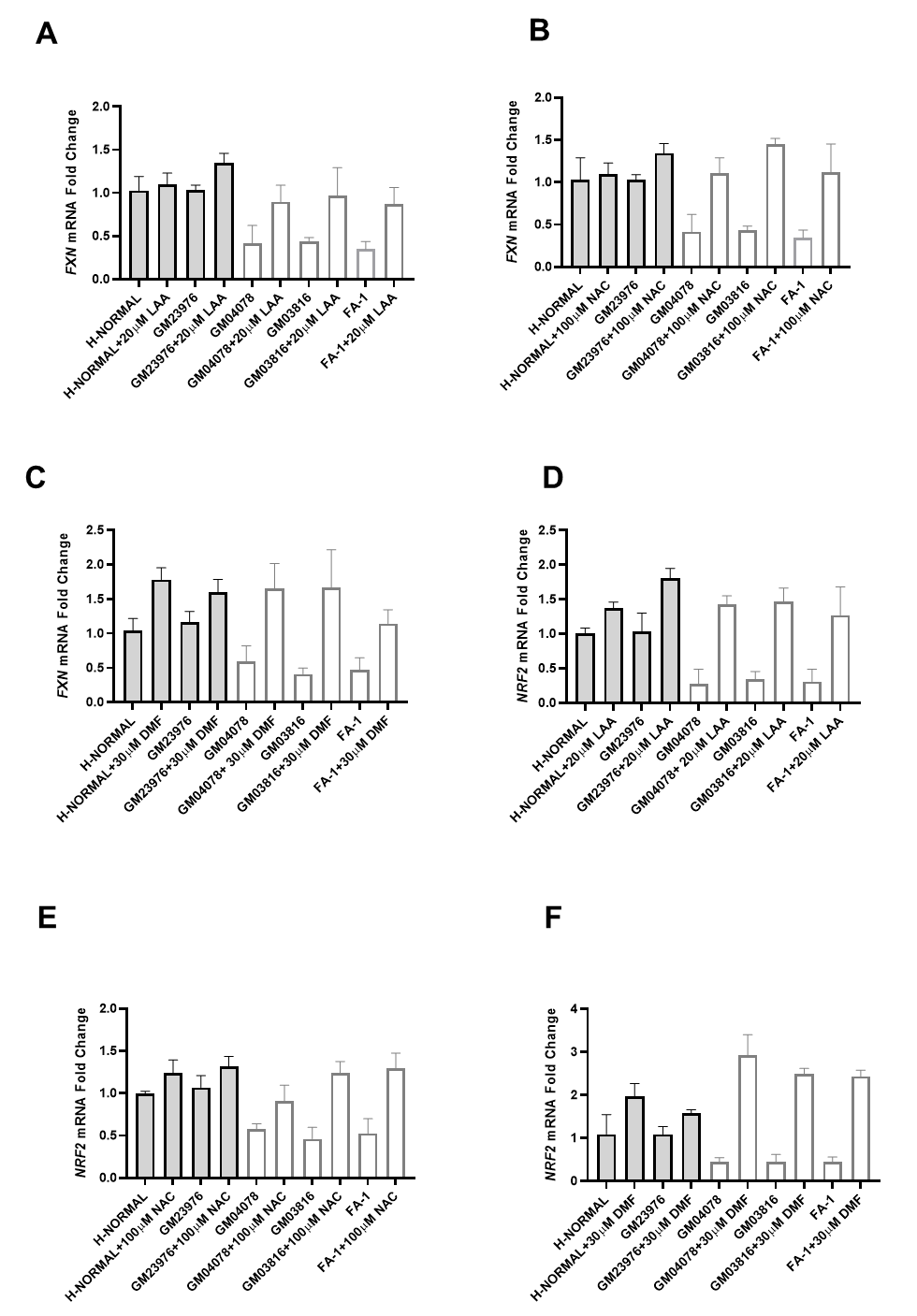
**

**Supplementary Figure 2. Treatment with LAA, NAC, and DMF led to significant increases in *FXN*and *NRF2*gene expression in human control and FRDA fibroblast cell lines.** Effect of (A) LAA, (B) NAC, (C) DMF treatments for 72 hours on *FXN*gene expression levels. Effect of (D) LAA, (E) NAC and (F) DMF treatments for 72 hours on *NRF2* gene expression levels. Five human fibroblasts cell lines were used, comprising two controls (H-Normal, GM23976) and three FRDA lines (GM04078, GM03816, FA-1). The mean value of all data set, from at least three independent experiments, normalised to the untreated groups of control (pegged at 1). All data are presented as mean±SEM.

**
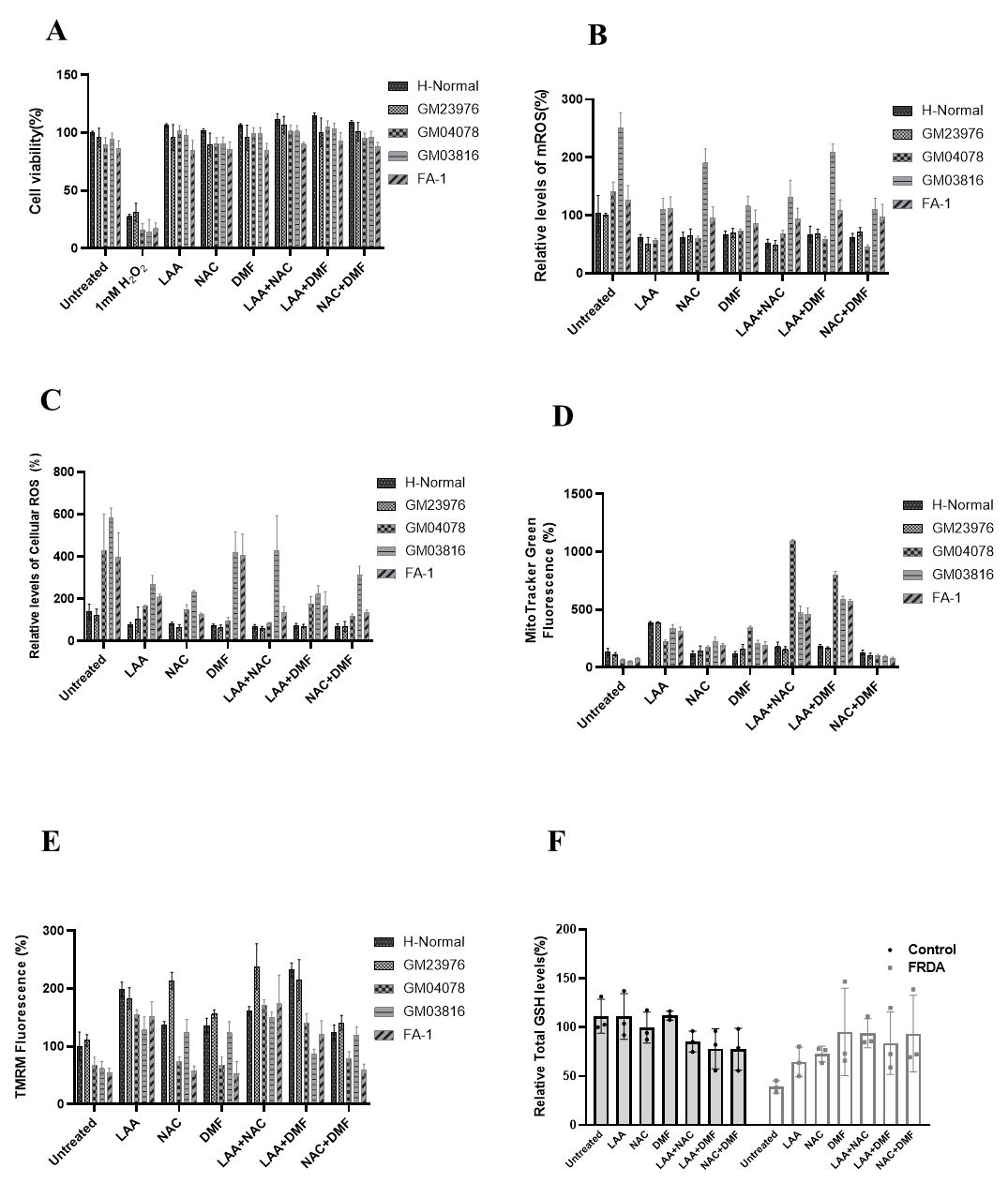
**

**Supplementary Figure 3. Effect of antioxidant combination on oxidative stress and antioxidant markers in human control and FRDA fibroblast cell lines.** (A) Protection against H_2_O_2_-induced oxidative toxicity by antioxidant combinations (20µM LAA, 100µM NAC and 30µM DMF) in human FRDA and control fibroblasts. Five human fibroblasts cell lines were used, comprising two controls (H-Normal, GM23976) and three FRDA lines (GM04078, GM03816, FA-1). Antioxidant combinations’ impact (20µM LAA, 100µM NAC and 30µM DMF) on (B) mROS, (C) cytoplasmic ROS, (D) mitochondrial mass, (E) ΔΨ_M_, and (F) Relative Total GSH. Five human fibroblasts cell lines were used, comprising two controls (H-Normal, GM23976) and three FRDA lines (GM04078, GM03816, FA-1). The mean value of all data set was normalised to the untreated group (set at 100%). All data are presented as mean±SEM from three independent experiments.

**
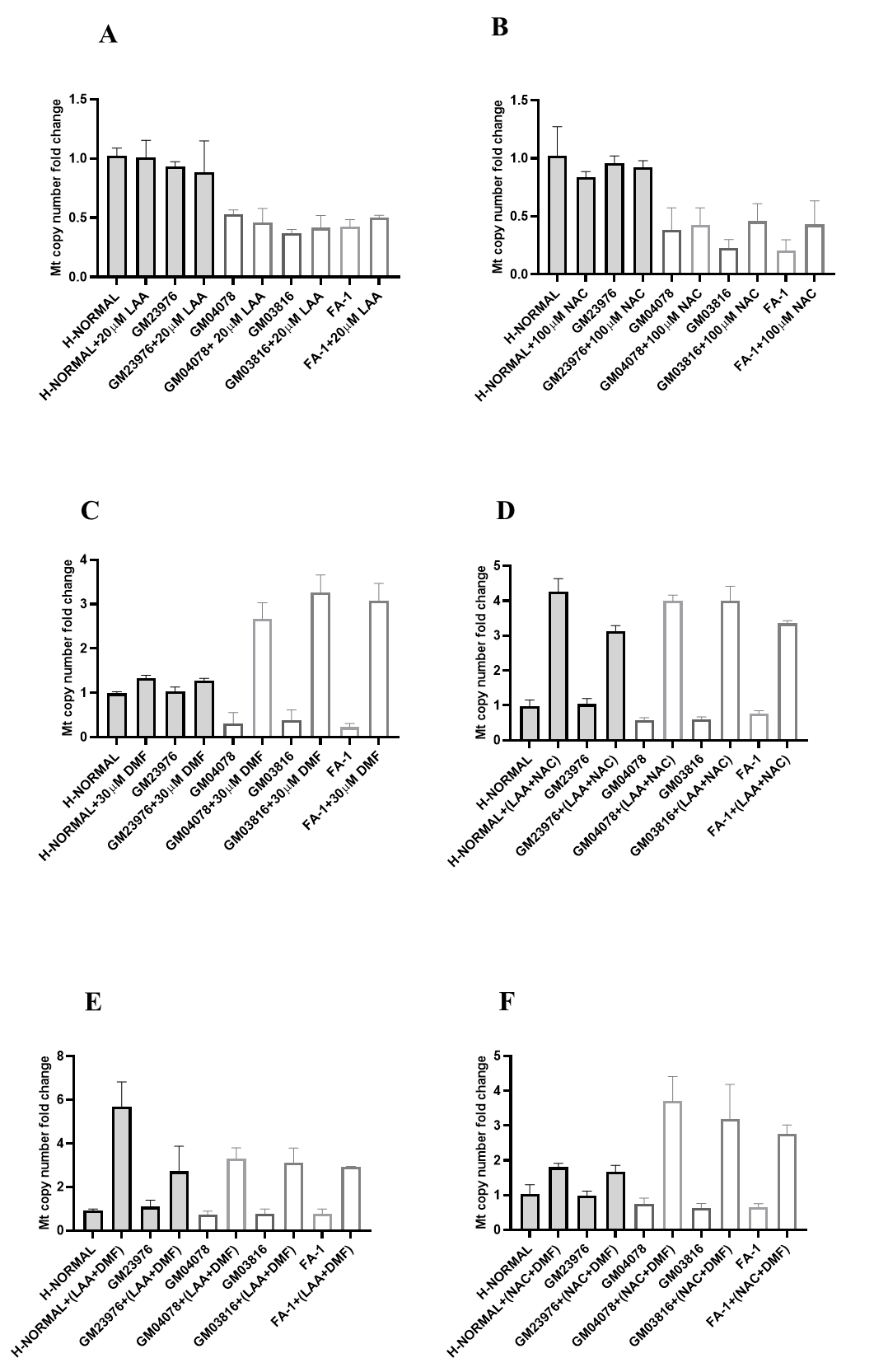
**

**Supplementary Figure 4. Mitochondrial DNA copy number analysis in human control and FRDA fibroblast cell lines.** qPCR analysis of mtDNA/nDNA ratio after 72 hours treatment with (A) LAA, (B) NAC, (C) DMF, (D) LAA+NAC, (E) LAA+DMF, and (F) NAC+DMF. Five human fibroblasts cell lines were used, comprising two controls (H-Normal, GM23976) and three FRDA lines (GM04078, GM03816, FA-1). The mean value of all data set, from three independent experiments, was normalised to the untreated groups of control (pegged at 1). All data are presented as mean±SEM.

 **Supplementary Figure 5. Effect of antioxidants on frataxin and NRF2 protein expression levels, aconitase activity, and Citrate synthase activity in human control and FRDA fibroblast cell lines.** Effect of antioxidants on (A) frataxin protein expression levels using ELISA, (B) NRF2 protein levels using ELISA, (C) aconitase activity, and (D) Citrate synthase activity. Five human fibroblasts cell lines were used, comprising two controls (H-Normal, GM23976) and three FRDA lines (GM04078, GM03816, FA-1). The mean value of all data set was normalised to the untreated group (set at 100%). All data are presented as mean±SEM from three independent experiments.

**Supplementary Figure 6. Effect of antioxidants on frataxin protein expression levels and NRF2 Translocation in human control and fibroblast cell lines.**  (A) Effect of LAA+NAC treatment on frataxin protein expression levels assessed by Western blot analysis. (B) Densitometry analysis of blots. A representative image analysis of NRF2 Translocation in (C) FRDA fibroblast cell line, (D) FRDA treated with NAC, and (E) FRDA treated with NAC+DMF, using Mender's coefficient with Image J software. An M1 value over 0.5 indicates strong colocalisation of Nrf2 with the DAPI signal, demonstrating strong nuclear colocalisation. Five human fibroblasts cell lines were used, comprising two controls (H-Normal, GM23976) and three FRDA lines (GM04078, GM03816, FA-1). The mean value of all data set was normalised to the untreated group (set at 100%). All data are presented as mean±SEM from three independent experiments.

**Supplementary Figure 7. Therapeutic effects of antioxidants in 2D sensory neurons.** Effect of LAA+NAC on (A) *FXN* and (B) *NRF2* gene expression levels. Data from three independent experiments were normalised to the untreated control group (set at 1). Effect of LAA+NAC on (C) frataxin and (D) NRF2 protein expression levels. Data from three independent experiments were normalised to the untreated control group (pegged at 100%). (E) Mitochondrial DNA copy number following qPCR analysis of mtDNA/nDNa ratio after 72 hours treatment with LAA+NAC. Data from three independent experiments were normalised to the untreated control group (set at 1). (F) Assessment of mROS using MitoSOX Red dye in 2D sensory neurons derived from human FRDA iPSCs after treatment with LAA+NAC for 72 hours. The mean value of all data set, from three independent experiments, was normalised to the untreated groups of control (pegged at 100%). All data are presented as mean±SEM. Four human iPSC-derived 2D sensory neurons lines were used, comprising two control (C3348, C6719) and two FRDA lines (F281, F4193).
